# A hole in the bucket? Exploring England’s retention rates of recently qualified GPs

**DOI:** 10.1186/s12960-025-00980-x

**Published:** 2025-03-03

**Authors:** William L. Palmer, Lucina Rolewicz, Victoria Tzortziou Brown, Giuliano Russo

**Affiliations:** 1 Nuffield Trust, London, UK; 2 Wolfson Institute of Population Health, Queen Mary University of London, London, UK

**Keywords:** Physicians, Family doctors, General practitioners, England, Education, Medical, Graduate, Labor supply, Health workforce

## Abstract

**Background:**

As the senior medics within primary care services, general practitioners (GPs) have a pivotal role within the National Health Service (NHS). Despite several commitments made by government to increase the number of GPs in England, the level has consistently fallen. Much attention has been paid to recruitment of trainee GPs and overall retention, whereas this study sought to examine the specific transition from ending training to joining the NHS.

**Methods:**

The study used aggregated, published administrative data to examine rates at which 14,302 doctors leaving their third year of specialty training (GP ST3s) became fully qualified GPs in NHS practices between 2018 and 2023. We separately analysed average levels of part-time working of those joining the NHS from 21,293 fully qualified joiners in England between 2017 and 2023. We calculated joiner and participation rates and used generalised linear mixed-effects models to explore possible demographic, period and cohort effects.

**Results:**

Of those doctors leaving their third year of training since 2018, around a third (34.3%) were recorded as having taken up a fully qualified GP role in NHS general practices 6 months after finishing training, rising to 47.5% within 1 year, and 62.2% within 2 years. Average estimated participation rates of joiners seemed to remain consistent at about 65–69% of a full-time contract between 2017 and 2023.

Joiner rates were lower for doctors with a primary medical qualification from outside the UK and, over a 2-year timeframe, both UK and non-UK trained male GP ST3s. Our statistical modelling suggests that there is a significant ‘period effect’ in connection to the recent Covid-19 pandemic, with apparent differences in the likelihood of GP ST3s joining the NHS in a fully qualified role at certain points in time, and an effect among some cohorts, with doctors who left specialty training in specific periods having significantly different joiner rates.

**Conclusion:**

The GP pipeline is expanding, but we find no evidence that retention of newly trained GPs is improving. We discuss possible factors for such attrition, from barriers to hiring new doctors, to their diminishing interest in joining the NHS. The data do not capture all destinations of GP ST3s, and more work is needed to further explore the changing career behaviours of subsequent cohorts and demographics of doctors completing GP training.

## Introduction and background

General practice is essential for population health, the efficiency of health systems, and ultimately the attainment of universal health coverage worldwide [1]. And as the senior medics within these services, general practitioners (known as family physicians in some countries) have a pivotal role, with responsibility for providing continuous whole person medical care, and managing risk, uncertainty and medical complexity [2, 3].

However, recruiting and retaining general practitioners (GPs) has historically been difficult [4]. The international literature suggests that worse pay, lower prestige, compulsory rural placements, elevated risk of burn-out, and a deteriorating working environment, might all be factors [5, 6]. Recent policies have mainly focused on increasing training numbers despite the recognition that workforce retention needs to be prioritised too [7].

Across the United Kingdom’s publicly funded National Health Service (NHS), the GP workforce appears to have been particularly affected by underinvestment in some areas, the Covid-19 pandemic, and the country’s aging population and shifting epidemiological profile [8]. The present paper is part of a special collection on the crisis of the medical workforce in Europe and offers a contribution to the exploration of aspects of the complex crisis of the GP workforce in the UK [9].

“General practitioner” is a protected title requiring specific professional registration to use. Becoming a GP in the UK typically takes a minimum of 10 years of medical training. Five of these years are usually in undergraduate medical education, 2 years are spent in the postgraduate UK Foundation Training programme and a minimum of an additional 3 years in GP specialty training [10]. GP training is open to UK medical graduates who have completed the foundation training; however, an increasing proportion of training places are also open to candidates from overseas. Indeed, the proportion of international medical graduates (IMGs) in GP training roles has risen, from 29% in December 2018 to 54% 5 years later. There has also been a rise in the proportion of doctors in GP training who are female (57% in 2018 compared to 65% in 2023) and aged 35 and over (31–39%) [11].

As of December 2023, there were around 6300 general practices across England—independent contractors, commissioned by the NHS—engaging approximately 27,000 fully qualified, full-time equivalent (FTE) GPs [11, 12]. Following their qualification, those GPs remaining in NHS general practice will usually practise either as independent contractors (or partners) running a practice, or as salaried GPs employed by a GP practice and/or as a locum GP filling in rota gaps (see Table 1). Recruitment to these roles is performed locally, as and when needed, by individual general practices. Other roles for those completing GP training include working in another country, either elsewhere within the UK or overseas, in other parts of the NHS such as in out-of-hours services, or providing private healthcare.

**Table 1:** Overview of key medical roles in general practice considered in our analysis

Role	Role description
GP partner	Self-employed practitioner that owns part of the practice and is subcontracted to provide services for the NHS. Provides both clinical sessions and business management
GP registrar	Doctors training to become general practitioners
GP regular locum	A practitioner that provides temporary cover in the absence of regular practitioners on a fairly predictable or consistent basis. Those working on less predictable patterns are referred to as ‘ad hoc locums’
Salaried GP	A contracted practitioner employed by a practice that receives a set salary
GP ST3	Doctors in their third year of specialty GP training
GP joiner	New doctors joining practices providing NHS services as fully qualified GPs after completing specialty training—this includes partners, salaried GPs and regular locums

*Source: Authors*

The general practice model in England requires sufficient GPs to act as partners, providing much of the organisational development capacity to meet the changing contractual, regulatory, and training requirements. It also requires enough GPs in general who can respond to the increasing demands of medical complexity in the community and can provide continuity of care [13]. If there are not enough GPs to provide and manage care, this is felt by patients struggling to book timely appointments, being unable to see their usual GP, and having a less positive experience of care overall. GPs are also required to supervise the wider clinical team.

There is a vast gap between ambition and reality in terms of numbers of GPs in England. This is despite several commitments made to increase the number of GPs in the last decade, including a pledge of 6000 more GPs by 2024 as part of the UK government’s 2019 election manifesto [14]. However, the number of fully qualified FTE GPs in England has been consistently falling, with a decrease of 1833 in the 7 years to December 2023 against the backdrop of a growing and aging population [15].

The government has set out ambitious plans to increase the number of GPs in England. The NHS Long Term Workforce Plan suggests that the number of GPs needs to increase by 39–47% (14–17,000) by 2036/2037 in order to meet demand and pledges to increase the number of GP training places by 50% by 2031/2032 [16]. The competition for GP training places has been increasing in recent years and there is an almost 100% fill rate of such places [17].

However, the efficiency of this training pipeline has been brought into question; previous work has suggested that on average, an estimated two training posts are required in England to get one fully qualified, FTE GP joining the NHS medical workforce (GP joiner) [18]. Such conversion rates of GP specialty trainees (GP registrars) into GP joiners are an important indicator of the efficiency of medical training. Previously surveys have found that 13% of GP registrars say they do not expect to work as GPs in the future and 60% do not report positive feelings about their future career prospects as a GP in the UK [19, 20], with career intentions adversely influenced by, for example, perceptions of workload pressure, low morale and poor work–life balance, as well as negative portrayals of general practice by politicians and the media [21].

## Methods

The aim of this paper is to describe the rate of GP registrars in their third year of training (GP ST3s) subsequently joining NHS general practice as fully qualified practitioners (GP joiners) in England, within the context of other physician roles in the NHS (see Table 1).

Drawing from the existing literature on GP registrars in the UK [19, 20], our starting hypothesis was that there is a substantial loss in workforce capacity caused by many doctors reaching the end of their training and then either not participating in NHS work, or, if joining the NHS workforce, not working full-time. We therefore set out to analyse the changing transition rates, from the third year of GP training to joining the fully qualified GP workforce, across the 5 years to 2023. The study covers two aspects of the transition of GP ST3s in NHS GP services:

1. The proportion of GP ST3s appearing in the NHS GP workforce dataset as fully qualified GPs working in partner, salaried or regular locum^1^ roles *(joiner rates)*; and
2. The average contracted hours of joiners, as a proportion of a typical full-time contract *(participation rates)*.

We first explored descriptively the available quantitative data; we then complemented descriptive statistics with statistical modelling to provide evidence of association between the components for any trends. We sought to explore the effects of different behaviours of new cohorts completing training (so called *cohort effect*) and the underlying change in landscape which might affect joiner and participation rates, including during the Covid-19 pandemic (*period effect*). We also explored the associations with the limited demographic variables available in the data (age, gender and country of qualification).

### Data sources

The data on joiner rates—used in the descriptive analysis and statistical modelling—were extracted from publicly available datasets published by NHS Digital, now part of NHS England, and were reported quarterly from September 2018 to December 2023. A dataset tracking 14,325 GP ST3s in England into fully qualified GP roles was used, with breakdowns by gender and country of qualification [22].

For participation rates, data on 21,293 fully qualified GP joiners in England were analysed, using the age and gender breakdowns of the GP ST3 practice-level data [12]. Data on participation rates were taken annually from September 2017 to December 2023 [12]. In this data, a full-time contract is defined as being 37.5 h.

### Data analysis

For the descriptive analysis (reported in Table 2), joiner rates—and relative risks—were calculated, disaggregated by cohort, gender and country of primary medical qualification. Participation rates among fully qualified practitioners joining the workforce (reported in Table 3) were calculated by dividing full-time equivalent by headcount number. These participation rates were adjusted (to reduce the influence of participation rates of those re-joiners rather than those directly from the domestic training pipeline) using GP ST3 age and gender breakdowns of those in their last year in a GP training post to estimate the average level of participation for each cohort joining the GP workforce from a third year training post.

**Table 2:** Joiner rates at 6 and 24 months after leaving training (unadjusted)

		Joined within 6 months			Joined within 24 months		
		Number leaving ≥ 6 months ago (%)	% identified in fully qualified NHS role (%)	Relative risk of joining (95% CI)	Number leaving ≥ 24 months ago (%)	% identified in fully qualified NHS role (%)	Relative risk of joining (95% CI)
All		13,601	34.3		8463	62.2	
Demographics							
UK trained	All	9220 (67.8%)	35.4	(ref)	6106 (72.1%)	65.4	(ref)
Non-UK trained	All	4381 (32.2%)	32.0	0.91 (0.86–0.95)	2357 (27.9%)	53.7	0.82 (0.79 –0.86)
All	Female	8288 (60.9%)	33.7	(ref)	5289 (62.5%)	64.0	(ref)
All	Male	5313 (39.1%)	35.3	1.05 (1.00–1.10)	3174 (37.5%)	59.1	0.92 (0.89–0.96)
UK trained	Female	6018 (44.2%)	35.2	(ref)	4043 (47.8%)	66.6	(ref)
	Male	3202 (23.5%)	35.7	1.02 (0.93–1.10)	2063 (24.4%)	63.1	0.95 (0.87–1.03)
Non-UK trained	Female	2270 (16.7%)	29.7	0.84 (0.76–0.93)	1246 (14.7%)	55.6	0.83 (0.75–0.93)
	Male	2111 (15.5%)	34.5	0.98 (0.89–1.08)	1111 (13.1%)	51.6	0.77 (0.69–0.87)
Year cohort left training							
Year to June 2019		2355 (17.3%)	34.1	(ref)	2355 (27.8%)	62.2	(ref)
Year to June 2020		2375 (17.5%)	39.4	1.15 (1.07–1.24)	2375 (28.1%)	66.9	1.08 (1.03–1.12)
Year to June 2021		2762 (20.3%)	35.7	1.04 (0.97–1.13)	2762 (32.6%)	60.5	0.97 (0.93–1.02)
Year to June 2022		2751 (20.2%)	31.7	0.93 (0.86–1.00)	971 (11.5%)	55.4^a^	0.89 (0.84–0.95)
Year to June 2023		3358 (24.7%)	31.8	0.93 (0.86–1.00)	n/a	n/a	n/a

^*a*^*Data cover cohort leaving ST3 in 6 months to December 2021*

**Table 3:** Frequency and participation rate of new fully qualified GPs joining the workforce by age, gender and year of joining, 2017–2018 to 2022–2023

	Numbers joining (headcount)	Average participation, % full-time contract	
All	21,293	64.1% (66.6% age-gender adjusted)	
Gender			
Female	13,258	60.9%	
Male	8,035	69.3%	
Age			
Under 30	1124	72.6%	
30–34	6993	66.4%	
35–39	5032	62.1%	
40–44	3170	61.8%	
45–49	2085	63.5%	
50–54	1320	64.8%	
55–59	880	62.9%	
60–64	428	54.2%	
65 and over	273	47.3%	
Year joined			
2017–2018	3291	64.8% (67.0% age-gender adjusted)	
2018–2019	4022	63.3% (66.1%)	
2019–2020	3787	62.3% (64.9%)	
2020–2021	3425	64.6% (67.7%)	
2021–2022	3075	66.8% (69.0%)	
2022–2023	3693	63.2% (65.8%)	

*Source: NHS England—General Practice Workforce (2024) *[12]

*Due to missing data values, some demographic breakdowns may not sum the total number of joiners. Age-gender adjustments to participation rates were based on the composition of ST3 GPs in the same year that the fully qualified GP joiner data relate to. Since most GPs will not take up a fully qualified role within a year of completing training, these rates should be interpreted with caution*

For the statistical modelling of joiner rates, we used generalised linear mixed-effects models to make statistical inferences about how our model predictors influenced the joiner rates in a given period. Our modelling framework is also known as a Hierarchical Age-Period-Cohort-Cross-Classified Random Effects Model, which can be used for data that are classified by age or time across multiple time periods and cohorts [24]. We included fixed effects for the time in months between GP ST3s last seen in specialty training and taking up a fully qualified GP role (referred to as ‘duration’), month of the year last seen in specialty training (to account for seasonality around reasons for leaving), and included random effects for cohort (the quarter and year in which doctors were last seen in specialty training) and period (the quarter and year at which doctors returned as a fully qualified GP) (Appendix 2, Equation 1). In some versions of the model (as flagged in the results), we also included fixed effects to capture demographic details around gender and country of qualification (UK and non-UK) (Appendix 2, Equation 2).

As the participation data did not track consistent cohorts over time, as with the joiner data, we were not able to conduct similar statistical modelling for this. Analysis was conducted in RStudio 4.3.2 and Microsoft Excel.

To quantify the relationship between the fixed effects and the likelihood of joining the fully qualified GP workforce, we presented odds ratios with 95% confidence intervals at the 5% significance level. We extracted the random effect components of our models, which were presented as conditional log odds with 95% confidence intervals for both the cohort and period effects (see Appendix 2 for further details on statistical modelling).

## Results

The results section starts with a description of the doctor population covered by the data, before giving a descriptive summary of variation and trends in joiner rates and then participation rates. We then cover the findings from the exploratory statistical modelling.

Across the 21 cohorts, between September 2018 and September 2023, 14,325 doctors left their final year of GP training (GP ST3s). Our findings show that the GP training pipeline is expanding; the number at the end of training increased over time with 2363 doctors leaving their final year of training in the year to June 2019 compared to 3358 in the corresponding period 4 years later. Around three-fifths (61.4%) of GP ST3s were female, and two-thirds (67.1%) gained their primary medical qualification in the UK with the remainder from European Economic Area (EEA) (4.4%) and elsewhere (28.5%).

**Fig. 1:**
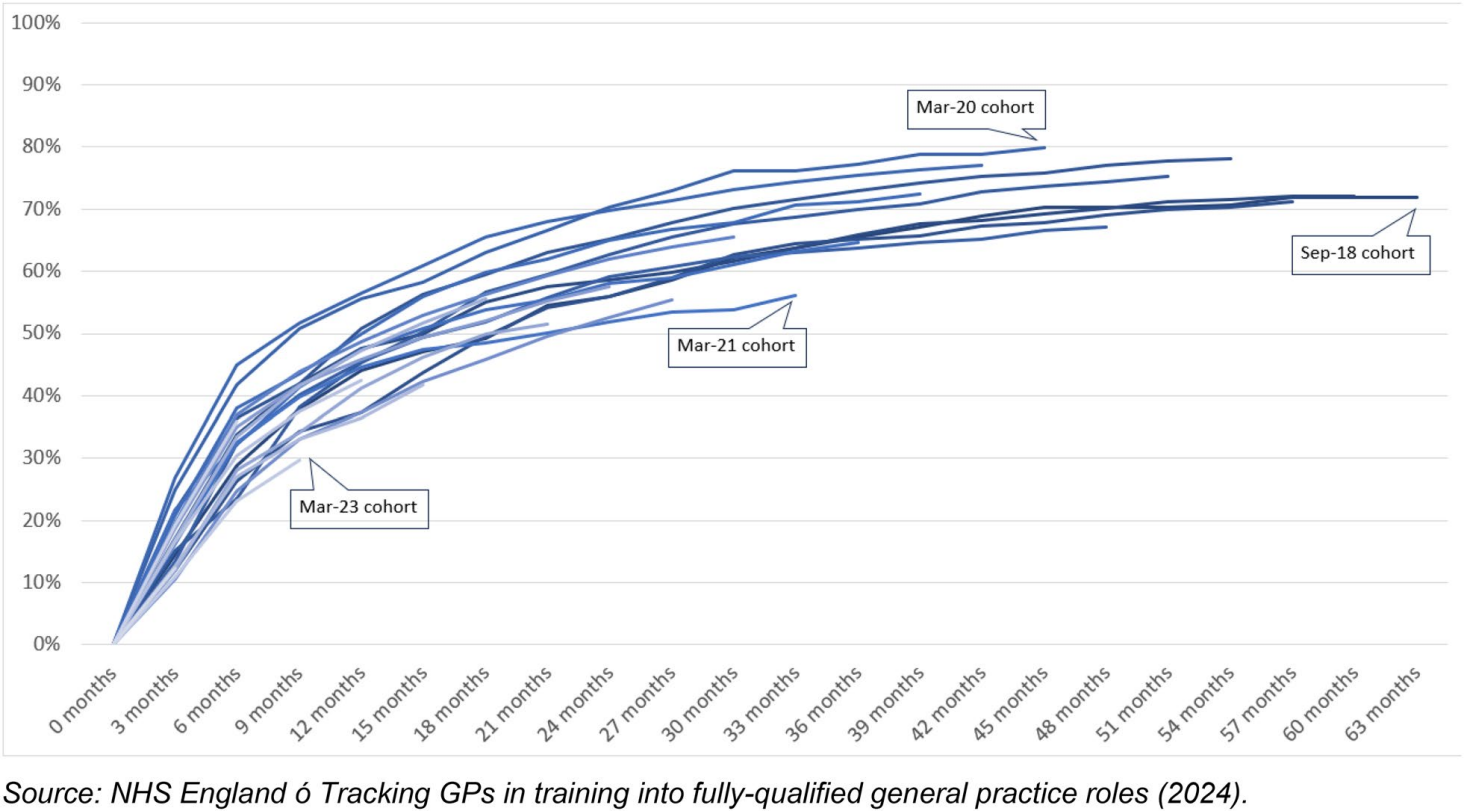
Progression rates of GPST3s joining the NHS GP workforce

Fewer than three-in-five (57.5%; 8237) of these doctors were identified in the NHS GP workforce subsequent to finishing training as a fully qualified GP by December 2023 while the remaining 6088 have yet to be identified in the data. By displaying the proportion who have joined the NHS in relation to months elapsed since that cohort left their ST3 year, we can visualise the variation in the likelihood of different GP ST3 cohorts joining at various times after leaving training (Fig. 1).

Excluding those recent cohorts for which insufficient time has elapsed for them to be captured within the timeframes, of those doctors in their third year of training since 2018, around a third (34.3%) were recorded as having taken up a fully qualified GP role in NHS general practices 6 months after finishing training rising to 47.5% within 1 year, 62.2% within 2 years, and 70.8% within 3 years.

These joiner rates were not consistent across cohorts or characteristics (Table 2). For example, overall doctors with a primary medical qualification from outside the UK were less likely to transition to fully qualified NHS GP roles within 6 months (− 3.4 percentage points; unadjusted relative risk (RR) 0.91, 95% CI (confidence interval) 0.86–0.95) with the disparity increasing over a 2-year period (− 11.7 percentage points; RR 0.82, CI 0.79–0.86).

Both UK and non-UK trained male GP ST3s were less likely to join within a 2-year timeframe compared to female GP ST3s (− 4.9 percentage points; RR 0.92, CI 0.89–0.96). Male non-UK trained GP ST3s seem to have the lowest overall joining rates at 2 years (RR 0.77, CI 0.69–0.87).

Since around 2021, there appears to have been a decline in the proportion of GP ST3s taking up fully qualified GP roles in NHS general practice services. We found 34.1% of GP ST3s in the year to June 2019 took up a fully qualified role within 6 months, with this proportion increasing for the cohort who left training the following year (39.4%, RR 1.15, CI 1.07–1.24) before declining. The recent two annual cohorts (those leaving in the year to June 2022 and to June 2023) appear to have generally lower joiner rates although these results were not always statistically significantly different to the baseline (year to June 2019). We present a visualisation of joiner rates disaggregated by region of qualification and gender in Fig. 4 in Appendix 1.

### Results from generalised linear mixed-effects models

**Fig. 2:**
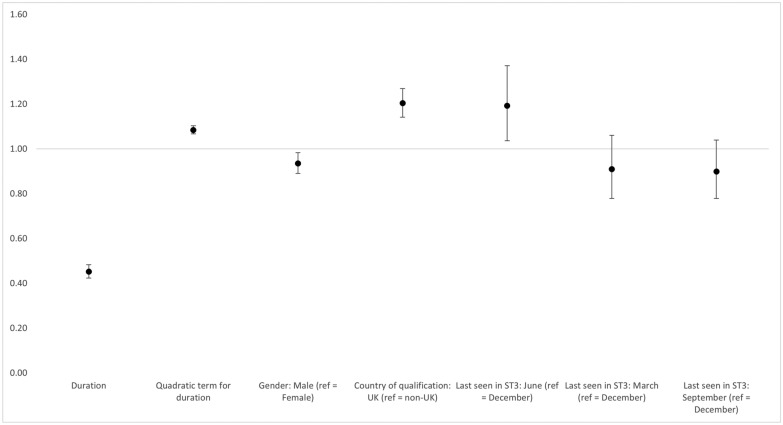
Odds ratios and confidence intervals of fixed effects from generalised linear mixed-effects model of joiner rates, adjusted for demographics, duration, seasonality, cohort and period

The findings from the statistical modelling support the previously described findings around associations between joiner rates and demographics. Nearly all of the fixed effects were significant predictors in how likely GP ST3s were to join the fully qualified NHS GP workforce. Male GP ST3s were less likely to join than female GP ST3s (odds ratio, OR 0.94, CI 0.89–0.98), while those who studied medicine in the UK were more likely to join than those who trained outside of the UK but took up a specialty training post in England (OR 1.20, CI 1.14–1.27) (Fig. 2).

We also found an association between when in the year GP ST3s left their training and their likelihood of joining the NHS. Relative to those last seen in their third year of specialty training in the 3 months to December, those last seen in 3 months to June were more likely to join as a fully qualified GP (OR 1.19, CI 1.04–1.37), but there was no significant difference in the likelihood of GP ST3s joining among those last seen in the 3 months to March (OR 0.91, CI 0.78–1.06) or September (OR 0.90, CI 0.78–1.04). As expected, our analysis also showed that the odds of joining increased at a decreasing rate as the length of time between training and acquiring a fully qualified role increased (OR 1.09, CI 1.07–1.10).

**Fig. 3:**
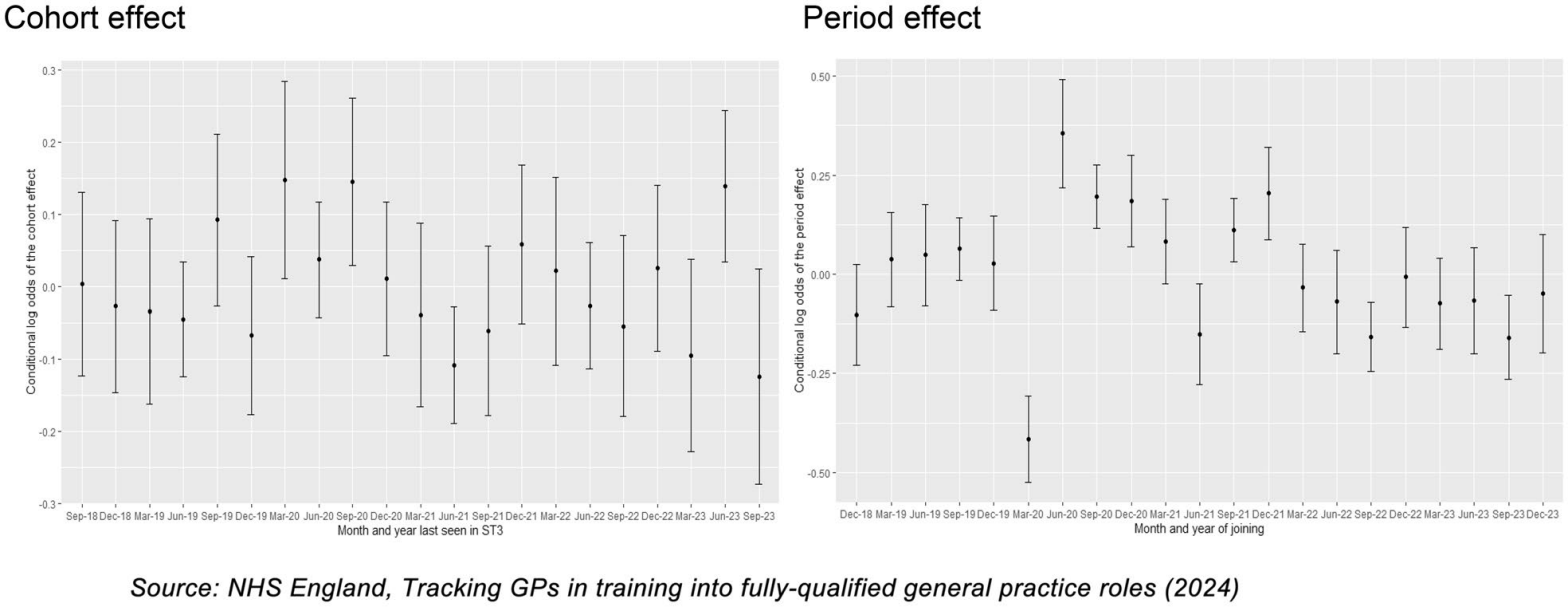
Likelihood (log odds) of GP ST3s joining the NHS fully qualified GP workforce by year of GP ST3 training, by month and year of leaving GP ST3 training (cohort effect) and by date of joining the NHS workforce (period effect), adjusting for duration, gender, country of qualification and seasonality

**Fig. 4:**
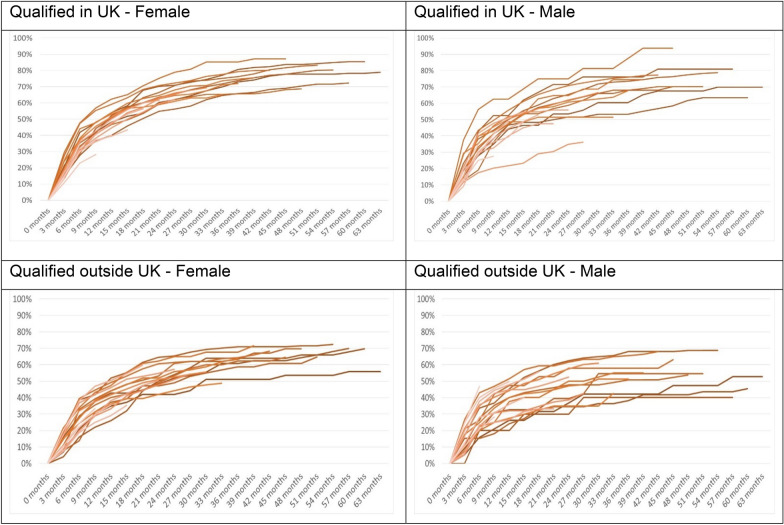
Progression rates of GP registrars nearing end of training (ST3) to becoming GPs employed directly in NHS general practice services

**Fig. 5:**
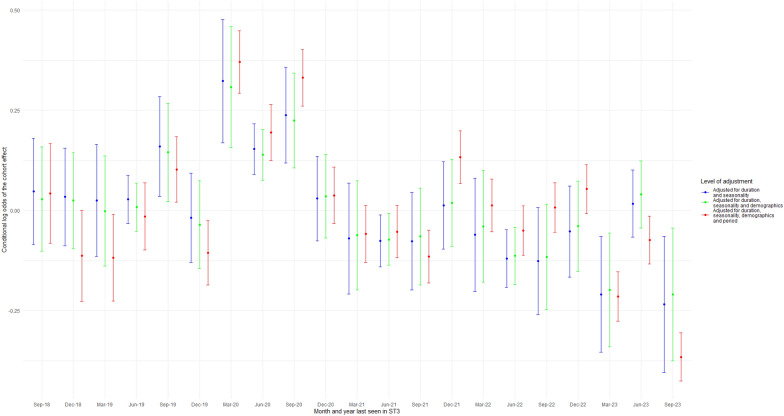
Log odds and confidence intervals from general linear mixed-effects model for the cohort effect

We identified a significant effect among some cohorts. After adjusting for duration and seasonality, GP ST3s who left specialty training in the 3 months to March 2020 were most likely to join the fully qualified workforce, while those who left specialty training in the 3 months to September 2023 were least likely to join (Fig. 5, Appendix 2). After adjusting for gender and country of qualification, some differences persisted including significantly lower joiner rates for the cohort leaving in the 3 months to June 2021 and higher rates among those last seen in specialty training in 3 months to March 2020 and to September 2020 (Fig. 3).

**Fig. 6:**
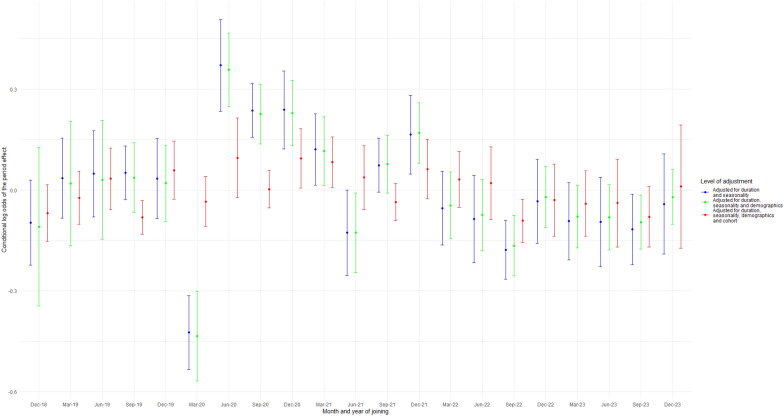
Log odds and confidence intervals from generalised linear mixed-effects model for the period effect

We also identified a significant ‘period effect’ with apparent differences in the likelihood of GP ST3s joining the NHS in a fully qualified role at a particular point in time. Specifically, after adjustments for duration and seasonality effects, doctors were least likely to join in the 3 months to March 2020 and most likely to join in the subsequent 3 months (to June 2020) (Fig. 6, Appendix 2). The differences persisted even after adjusting for demographic factors for gender and country of qualification (Fig. 3).

### Participation rates

As well as joiner rates, we also explored differences in ‘participation rate’ which relates to the contracted hours of joiners. In the year to December 2023, the average participation—the extent to which staff are employed on a full-time contract—of fully qualified GP joiners was 64%. When breaking participation down by 5-year age category among those who joined between 2017–2018 and 2022–2023, participation varied from 47% among GPs aged 65 and over to 73% among those under 30. Average participation rates are lower for female GPs (61%).

If it is assumed that new GP joiners follow the age and gender distribution of those in their third year of GP training, then the participation rate would be in the region of 66% in 2022–2023. These levels do vary to a degree from year-to-year, but were similar to those seen in the baseline year, 5 years’ prior (67%) (Table 3).

## Discussion

While the GP workforce equation has often been characterised in the literature as a ‘recruitment or retention crisis’ [16, 23, 24], our paper focuses on the critical issue of conversion of GP training numbers to NHS GP joiners in England. The leak in the pipeline from training through to participation in the NHS workforce as fully qualified GPs has been highlighted previously [18]; exploiting newly published data, we explored the nature of the challenge in greater depth.

Our analysis of GP ST3s’ NHS career progression suggests a substantial attrition rate in the GP workforce model; of those doctors in their final stage of training since 2018, fewer than two-thirds (62.2%) were captured in the data as having taken up a fully qualified GP role in NHS general practice within 2 years. This trend in recent years appears to have worsened, as little more than half (55.4%) of those that left their third year of training between June and December 2021 had taken up fully qualified NHS GP roles within 2 years and by December 2023. Some demographics display especially low joiner rates, particularly males with a primary medical qualification from outside the UK, of which only 51.6% leaving their third year of training appear to take up fully qualified roles in NHS general practice within 2 years.

Our exploratory statistical analysis also provides some evidence that certain cohorts of GP ST3s were more likely to join as fully qualified GPs (such as those that left in 2020), and that at certain periods of time newly qualified doctors were more likely to be recruited. For example, the 3 months to June 2020 saw significantly higher joiner rates relative to average, though this may be an artefact of Covid-19 with a catch-up in recruitment after lower rates in the previous 3 months as the pandemic first hit.

The impact of these low joiner rates on overall NHS capacity are compounded by relatively high but fairly stable levels of less than full-time working. The average contracted hours of GPs joining the workforce are around two-thirds of a full-time contract. Rates are lower for female GPs (61%), and broadly decline by age. Taking the joiner rates and participation rates together, it means that, for every 10 doctors leaving the third year of GP training, NHS general practices will secure around 4 full-time equivalent fully qualified GPs within 2 years, excluding those that might be working as ad hoc locums.

Our findings need to be interpreted with a degree of caution, as we acknowledge data limitations, and our results will need to be triangulated with other workforce data sources. For example, our analysis did not include ad hoc GP locums within the NHS because this group were not captured within the data employed in our work. While separate analyses suggest that around a third of GP ST3 leavers not taking up substantive NHS GP roles may have worked as an ad hoc locum this represents a small proportion of the overall GP workforce capacity (an estimated 418 FTE in December 2023) [11, 12, 22, 25]. The data do also not pick up, for instance, GPs working exclusively in certain settings such as out-of-hours services, working for NHS 111 (a digital triage service), A&E streaming or through an NHS-commissioned digital provider [22]. This may have underestimated conversion rates to GP roles but given the importance of—and current challenges around—a sustainable supply of substantive GP roles in NHS general practices, the rates we present remain of significant policy importance.

Our reported participation rates may also be affected by this lack of information on, for example, ad hoc locum sessions. The apparent level of participation is also sensitive to the definition used for ‘full-time’ particularly whether they are based on contracted hours or number of sessions, which are assumed to each be 4 hours 10 minutes but commonly are, in reality, longer. One study found that, depending on definition used, the proportion of GPs working at least full-time could be as high as 81.5% (based on the Office for National Statistics definition of full-time as 30 h a week) or 9.5% (based on the British Medical Association of 9 sessions); however, across all definitions, the proportion of GPs contracted full-time fell [25, 26].

We consider that several supply as well as demand-side factors might be responsible for such delays in joining the primary care medical workforce. On the one hand, it is fairly established that GP jobs have become increasingly complex and demanding, particularly after the recent pandemic [27]. A questionnaire of 25 European countries in 2015 showed that nations where GPs undertook more than 25 direct patient consultations per day experienced more problems in GP retention and recruitment [28].

The definition of clinical sessions in existing NHS GP contracts and the implementation of such contracts often underestimate the actual GP workload, with average hours per session typically far exceeding the assumed length and this having increased over time [25, 27]. By experiencing their tutors’ workload, it does not seem unreasonable that trainees may be put off from pursuing a full-time career in general practice [19]. Similarly, doctors might delay committing to a substantive GP role where contracts do not sufficiently capture additional, non-clinical activities such as education, management and research which may be better captured within secondary care specialist consultant contracts.

At the same time, there are reports it might have become increasingly difficult for GP practices to contract newly trained GPs because of funding shortfalls at different levels [29]. The introduction of the Additional Roles Reimbursement Scheme (ARRS) in 2019 meant that practices could benefit from additional staff at no direct cost to their practice and resulted in an additional 22,000 staff being recruited. The ARRS scheme however did not, at the time of writing, include salaried GPs. Therefore, the relative costs to practices of employing GPs combined with wider financial pressures within general practice may have resulted in fewer GP job opportunities [30].

Our work carries important policy implications for governments in the UK as well as in other European countries. First of all, if one-third of recently trained specialists these days fail to join the general practice workforce in salaried, partner or regular locum roles, this likely means that more GPs need to be trained or retained to meet the increasing health needs and demand for healthcare services from an aging population, exacerbating an already complex planning exercise [31].

Secondly, the root causes for the apparent leak need to be fully understood and tackled, with a view to adequately staffing primary care services. We identified a need for additional information on the variety and intensity of roles that GPs undertake within the NHS, as well on vacancies (both filled and unfilled) which is particularly problematic in light of recent reports of GPs being made redundant or unable to find work [16, 32]. Such data would allow the necessary analyses to gain a greater understanding of the nature and reasons for joiner and participation rates. Government has sought to attract GPs into the NHS through a range of schemes including GP International Induction Programme, improved visa sponsorship arrangements, GP Fellowship Programme, the Supporting Mentors Scheme and the New to Partnership Payment [18]. However, the continuation of funding for several of these initiatives is uncertain. Some areas find it more challenging to attract GPs. These are typically more deprived and remote locations that are not regularly used for medical school placements and are therefore less familiar to newer doctors [33].

The findings of particularly low levels of overseas doctors completing GP training being captured in the data as going on to take up fully qualified GP roles in NHS general practices is particularly important in view of the increasing proportion of IMG trainees. Other research has highlighted the range of psychological, social, and practical challenges faced by early career IMG GPs [34], which may contribute to them taking up the sort of roles, such as ad hoc locums or with digital providers, which are not captured as destinations in the data used in this study. Induction and training support schemes are attempting to address the differential exam attainment of IMGs in the GP licensing exams [35], and policies have been introduced to remove potential barriers to IMG GP trainees joining the NHS workforce once they have completed their training. The effectiveness of such initiatives needs ongoing monitoring.

## Conclusion

As the senior medics within their services, GPs have a pivotal role for the sustainability of the primary care system. However, in England there have been repeated failures to meet the ambitions to increase the number of fully qualified GPs and, instead, the promised increases have not materialised. Much attention has been paid to the recruitment of trainee GPs; however, our analysis shines a light on a critical part of the workforce model namely the domestic training pipeline and, specifically, the transition from ending training to joining NHS general practices.

Using published data, we estimated that for every 10 doctors leaving training around 4 fully qualified full-time equivalent GPs join NHS general practices in partner, salaried or regular locum roles within 2 years. Although this is based on contracted rather than actual hours worked and is, as outlined above, sensitive to the definitions used. We also provide evidence of differences between demographics (particularly lower joiner rates for overseas trained medics), differences in behaviours of doctors leaving over time (cohort effect), and different labour market conditions over time (period effect).

Further work is also suggested. The differences in career behaviours between demographics appear to be changing over time, but we were limited to characteristics available in the data (country of qualification, age and gender) and this should be studied further. An understanding of the effect of external conditions, such as alternative employment conditions at national and local levels, should also be sought. Conversion rates for GPs should be compared to rates for other specialties, to identify whether this issue is unique to general practice or is more common across other areas of medicine. Better data are also needed, particularly capturing actual hours worked by GPs and on the full range of destinations of GP ST3s. Given the importance of GP retention to the sustainability of GP services, national and regional bodies must act fast to better understand the factors influencing this apparent suboptimal transition from training to joining the NHS workforce and identify and implement urgent solutions.

## Data Availability

Data are available at: https://digital.nhs.uk/data-and-information/publications/statistical/general-and-personal-medical-services/31-december-2023.

## Appendix 1

See Fig. 4 .

## Appendix 2: further details on the statistical modelling

We used the analysis of variance (ANOVA) function to assess the fit of different mixed-effects models. This compared the fully adjusted model to each of the partially adjusted models using the Bayesian information criterion (BIC), where lower BIC values indicate a better fit of the model to the data.

The results in Table 4 below suggest that relative to the partially adjusted models, the fully adjusted model significantly reduces the residual sum of squares and is an improved fit over the other models.

**Table 4:** Associated ANOVA parameters of fully adjusted model relative to partially adjusted models

Model adjustment	Bayesian information criterion (BIC) for model adjusted for demographics, duration, seasonality, cohort and period	Bayesian information criterion (BIC)	*p*-value
Adjusted for duration, seasonality and period	3868.0	3928.2	< 0.001
Adjusted for duration, seasonality and cohort		4013.8	< 0.001
Adjusted for demographics, duration, seasonality and period		3882.1	< 0.001
Adjusted for demographics, duration, seasonality and cohort		3964.8	< 0.001

### Generalised mixed effects model specifications

1 $${\text{Logit}}\left( J \right) = \beta_{0} + \left( {\beta_{1} D} \right) + \left( {\beta_{2} D^{2} } \right) + \left( {\beta_{3} M} \right) + u_{C} + v_{P}$$

2 $${\text{Logit}}\left( J \right) = \beta_{0} + \left( {\beta_{1} D} \right) + \left( {\beta_{2} D^{2} } \right) + \left( {\beta_{3} M} \right) + \left( {\beta_{4} G} \right) + \left( {\beta_{5} Q} \right) + u_{C} + v_{P} ,$$

where *J* = proportion of joiners; *β*_*0*_ = intercept; *β*_*i*_ = fixed effects coefficients; *D* = duration; *M* = month of the year last seen in specialty training; *G* = gender; *Q* = country of qualification; _*C*_ = cohort and _*P*_ = period. Denominator-based weights were applied to adjust each group’s contribution to the overall parameter estimation.

### Sensitivity analyses

To understand whether there was any significant interaction between the gender and country of primary medical qualification of GP ST3s and the likelihood of joining the fully qualified workforce, we added an interaction term in the fully adjusted model as an additional fixed effect, but there was no significant association (OR 0.92, CI 0.83–1.02).

We also ran another model including all fixed and random effects as before, but excluding those who joined the fully qualified workforce after 42 months to see if this made any difference to the period effect, given the small number of observations in the data that joined after this point. Doctors were still least likely to join in March 2020 (LO − 0.403, CI − 0.514–− 0.293) and most likely to join in June 2020 (LO 0.381, CI 0.245–0.517), with little difference in the log odds relative to the fully adjusted model that included all observations.

We ran a further model using the cumulative proportion of joiners for each cohort as our outcome variable (red data series in Figs. 5  and 6), including random effects for both cohort and period. This showed a stronger effect among doctors who left specialty training between January and September 2020, who had a higher likelihood of joining the fully qualified GP workforce, whereas those who left specialty training between January and September 2023 were less likely to join (Fig. 5). When analysing the period effect, there was no difference in the likelihood of joining as a fully qualified GP in the 3 months to March 2020 and the 3 months to June 2020 (Fig. 6).

### Cohort effect

Doctors last seen in their third year of specialty training in the 3 months to March 2020 were more likely to join as a fully qualified GP compared to the average across all doctors who completed training between 2018 and 2023 (LO 0.323, CI 0.169–0.477). After further adjusting for gender and country of qualification, doctors who left specialty training in the 3 months to March 2020 were still more likely to join the fully qualified workforce (LO 0.308, CI 0.156–0.460). This effect remained when adding in the random effect for period to the model (LO 0.148, CI 0.011–0.285).

GP ST3s who were last seen in the 3 months to September 2023 were less likely to join as a fully qualified GP when only adjusting for duration and seasonality (LO − 0.234, CI − 0.404 – − 0.064). This difference remained when adjusting for gender and country of qualification (LO − 0.210, CI − 0.375– − 0.044), but was not statistically significant when accounting for the random effect for period (LO − 0.124, CI − 0.273–0.025).

### Period effect

Doctors who joined the fully qualified GP workforce were most likely to join in the 3 months to June 2020 across all doctors who joined between December 2018 and December 2023 (LO 0.370, CI 0.233–0.507), and least likely to join in the 3 months to March 2020 (LO − 0.424, CI − 0.533–− 0.315). After further adjusting for gender and country of qualification, doctors were still most likely to join the fully qualified workforce in the 3 months to June 2020 (LO 0.357, CI 0.248–0.467) and least likely to join in the 3 months to March 2020 (LO − 0.435, CI − 0.568–− 0.301).

These effects remained when adding in the random effect for cohort to the model (3 months to June 2020: LO 0.355, CI 0.219–0.492; 3 months to March 2020: LO − 0.416, CI − 0.525–− 0.307).

## Abbreviations

FTE: Full-time equivalent
GP ST3: Doctor in third year of GP specialty training
GP: General practitioner
IMGs: International medical graduates
NHS: National Health Service in England

## Footnotes

1 We did not include ‘ad hoc’ GP locums (locum or sessional GPs who typically work briefly at practices, such as a single one-off session, to cover for short-term or unexpected absences), because information about them is captured in a different way to the rest of the workforce.
